# Supplementary material for: Discovery of Pathogenic Variants Associated with Idiopathic Recurrent Pregnancy Loss Using Whole-Exome Sequencing
Source: Int J Mol Sci. 2024 May 17;25(10):5447. doi: 10.3390/ijms25105447 (PMC11121708; doi:10.3390/ijms25105447)
Supplement: Supplementary file 1 [file ijms-25-05447-s001.zip › ijms-2993324-supplementary.pdf]

Supplementary Table S1. List of abortion/miscarriage related genes

gene symbol	NCBI entrez ID	Gene Name
ABCC6	368	ATP binding cassette subfamily C member 6(ABCC6)
ABHD17B	51104	abhydrolase domain containing 17B, depalmitoylase(ABHD17B)
ACE	1636	angiotensin I converting enzyme(ACE)
ACE2	59272	angiotensin converting enzyme 2(ACE2)
ACER2	340485	alkaline ceramidase 2(ACER2)
ACKR4	51554	atypical chemokine receptor 4(ACKR4)
ACO1	48	aconitase 1(ACO1)
ACP1	52	acid phosphatase 1(ACP1)
ADAMTSL1	92949	ADAMTS like 1(ADAMTSL1)
AGTPBP1	23287	ATP/GTP binding carboxypeptidase 1(AGTPBP1)
AGTR1	185	angiotensin II receptor type 1(AGTR1)
AHR	196	aryl hydrocarbon receptor(AHR)
ALB	213	albumin(ALB)
ALDH1A1	216	aldehyde dehydrogenase 1 family member A1(ALDH1A1)
ALDH1B1	219	aldehyde dehydrogenase 1 family member B1(ALDH1B1)
ALPG	251	alkaline phosphatase, germ cell(ALPG)
ANKRD18A	253650	ankyrin repeat domain 18A(ANKRD18A)
ANKRD18B	441459	ankyrin repeat domain 18B(ANKRD18B)
ANKRD20A1	84210	ankyrin repeat domain 20 family member A1(ANKRD20A1)
ANKS6	203286	ankyrin repeat and sterile alpha motif domain containing 6(ANKS6)
ANP32B	10541	acidic nuclear phosphoprotein 32 family member B(ANP32B)
ANXA1	301	annexin A1(ANXA1)
ANXA5	308	annexin A5(ANXA5)
AOPEP	84909	aminopeptidase O (putative)(AOPEP)
APBA1	320	amyloid beta precursor protein binding family A member 1(APBA1)
APOE	348	apolipoprotein E(APOE)
APTX	54840	aprataxin(APTX)
AQP3	360	aquaporin 3 (Gill blood group)(AQP3)
AQP7	364	aquaporin 7(AQP7)
AR	367	androgen receptor(AR)
ARHGEF39	84904	Rho guanine nucleotide exchange factor 39(ARHGEF39)
ARID3C	138715	AT-rich interaction domain 3C(ARID3C)
ARNT	405	aryl hydrocarbon receptor nuclear translocator(ARNT)
ASPH	444	aspartate beta-hydroxylase(ASPH)
ASPN	54829	asporin(ASPN)
ATOSB	80256	atos homolog B(ATOSB)
AUH	549	AU RNA binding methylglutaconyl-CoA hydratase(AUH)
B4GALT1	2683	beta-1,4-galactosyltransferase 1(B4GALT1)

BAG1	573	BAG cochaperone 1(BAG1)
BANCR	100885775	BRAF-activated non-protein coding RNA(BANCR)
BARX1	56033	BARX homeobox 1(BARX1)
BICD2	23299	BICD cargo adaptor 2(BICD2)
BIN1	274	bridging integrator 1(BIN1)
BNC2	54796	basonuclin 2(BNC2)
C11ORF80	79703	chromosome 11 open reading frame 80(C11orf80)
C9ORF131	138724	chromosome 9 open reading frame 131(C9orf131)
C9ORF153	389766	chromosome 9 open reading frame 153(C9orf153)
C9ORF24	84688	chromosome 9 open reading frame 24(C9orf24)
C9ORF40	55071	chromosome 9 open reading frame 40(C9orf40)
C9ORF57	138240	chromosome 9 open reading frame 57(C9orf57)
C9ORF64	84267	Q-nucleotide N-glycosylase 1(QNG1)
C9ORF72	203228	C9orf72-SMCR8 complex subunit(C9orf72)
C9ORF85	138241	chromosome 9 open reading frame 85(C9orf85)
CA9	768	carbonic anhydrase 9(CA9)
CAAP1	79886	caspase activity and apoptosis inhibitor 1(CAAP1)
CARD19	84270	caspase recruitment domain family member 19(CARD19)
CARNMT1	138199	carnosine N-methyltransferase 1(CARNMT1)
CARNMT1-AS1	101927380	CARNMT1 antisense RNA 1(CARNMT1-AS1)
CBWD5	445571	Zn regulated GTPase metalloprotein activator 1C(ZNG1C)
CBWD5	220869	Zn regulated GTPase metalloprotein activator 1E(ZNG1E)
CBX3	11335	chromobox 3(CBX3)
CCDC103	388389	coiled-coil domain containing 103(CCDC103)
CCDC107	203260	coiled-coil domain containing 107(CCDC107)
CCDC171	203238	coiled-coil domain containing 171(CCDC171)
CCDC180	100499483	coiled-coil domain containing 180(CCDC180)
CCDC39	339829	coiled-coil domain containing 39(CCDC39)
CCDC40	55036	coiled-coil domain containing 40(CCDC40)
CCDC65	85478	coiled-coil domain containing 65(CCDC65)
CCDC68	80323	coiled-coil domain containing 68(CCDC68)
CCIN	881	calicin(CCIN)
CCL19	6363	C-C motif chemokine ligand 19(CCL19)
CCL21	6366	C-C motif chemokine ligand 21(CCL21)
CCL27	10850	C-C motif chemokine ligand 27(CCL27)
CCNB3	85417	cyclin B3(CCNB3)
CCNO	10309	cyclin O(CCNO)
CD163	9332	CD163 molecule(CD163)
CD164	8763	CD164 molecule(CD164)
CD226	10666	CD226 molecule(CD226)
CD68	968	CD68 molecule(CD68)

CD69	969	CD69 molecule(CD69)
CD7	924	CD7 molecule(CD7)
CD72	971	CD72 molecule(CD72)
CD82	3732	CD82 molecule(CD82)
CD84	8832	CD84 molecule(CD84)
CD8A	925	CD8 subunit alpha(CD8A)
CDC14B	8555	cell division cycle 14B(CDC14B)
CDK20	23552	cyclin dependent kinase 20(CDK20)
CDKN2A	1029	cyclin dependent kinase inhibitor 2A(CDKN2A)
CDKN2B	1030	cyclin dependent kinase inhibitor 2B(CDKN2B)
CDKN2B-AS1	100048912	CDKN2B antisense RNA 1(CDKN2B-AS1)
CEACAM1	634	CEA cell adhesion molecule 1(CEACAM1)
CEACAM5	1048	CEA cell adhesion molecule 5(CEACAM5)
CEACAM6	4680	CEA cell adhesion molecule 6(CEACAM6)
CEMIP2	23670	cell migration inducing hyaluronidase 2(CEMIP2)
CENPH	64946	centromere protein H(CENPH)
CENPP	401541	centromere protein P(CENPP)
CEP78	84131	centrosomal protein 78(CEP78)
CER1	9350	cerberus 1, DAN family BMP antagonist(CER1)
CER1	23739	chromosome 3 common eliminated region 1(C3CER1)
CFAP298	56683	cilia and flagella associated protein 298(CFAP298)
CFAP300	85016	cilia and flagella associated protein 300(CFAP300)
CFAP95	138255	cilia and flagella associated protein 95(CFAP95)
CHIA	27159	chitinase acidic(CHIA)
CHMP5	51510	charged multivesicular body protein 5(CHMP5)
CKS2	1164	CDC28 protein kinase regulatory subunit 2(CKS2)
CLTA	1211	clathrin light chain A(CLTA)
CNTFR	1271	ciliary neurotrophic factor receptor(CNTFR)
CNTLN	54875	centlein(CNTLN)
CNTNAP3	79937	contactin associated protein family member 3(CNTNAP3)
CNTNAP3B	728577	contactin associated protein family member 3B(CNTNAP3B)
COL13A1	1305	collagen type XIII alpha 1 chain(COL13A1)
COL1A1	1277	collagen type I alpha 1 chain(COL1A1)
COL1A2	1278	collagen type I alpha 2 chain(COL1A2)
COL4A6	1288	collagen type IV alpha 6 chain(COL4A6)
COL5A1	1289	collagen type V alpha 1 chain(COL5A1)
COL5A2	1290	collagen type V alpha 2 chain(COL5A2)
COL6A1	1291	collagen type VI alpha 1 chain(COL6A1)
COL6A3	1293	collagen type VI alpha 3 chain(COL6A3)
COL9A2	1298	collagen type IX alpha 2 chain(COL9A2)
CORO2A	7464	coronin 2A(CORO2A)

CREB3	10488	cAMP responsive element binding protein 3(CREB3)
CRISP3	10321	cysteine rich secretory protein 3(CRISP3)
CSF3	1440	colony stimulating factor 3(CSF3)
CSRNP3	80034	cysteine and serine rich nuclear protein 3(CSRNP3)
CTSL	1514	cathepsin L(CTSL)
CTSV	1515	cathepsin V(CTSV)
CXCL10	3627	C-X-C motif chemokine ligand 10(CXCL10)
CYP1A1	1543	cytochrome P450 family 1 subfamily A member 1(CYP1A1)
CYP1B1	1545	cytochrome P450 family 1 subfamily B member 1(CYP1B1)
CYP24A1	1591	cytochrome P450 family 24 subfamily A member 1(CYP24A1)
DAPK1	1612	death associated protein kinase 1(DAPK1)
DAPK1-IT1	100506897	DAPK1 intronic transcript 1(DAPK1-IT1)
DCAF10	79269	DDB1 and CUL4 associated factor 10(DCAF10)
DCAF12	25853	DDB1 and CUL4 associated factor 12(DCAF12)
DCTN3	11258	dynactin subunit 3(DCTN3)
DDX58	23586	RNA sensor RIG-I(RIGI)
DENND4C	55667	DENN domain containing 4C(DENND4C)
DHFR	1719	dihydrofolate reductase(DHFR)
DHFR	573971	dihydrofolate reductase pseudogene 1(DHFRP1)
DIAPH2-AS1	10824	DIAPH2 antisense RNA 1(DIAPH2-AS1)
DIRAS2	54769	DIRAS family GTPase 2(DIRAS2)
DMRTA1	63951	DMRT like family A1(DMRTA1)
DNAAF1	123872	dynein axonemal assembly factor 1(DNAAF1)
DNAAF11	23639	dynein axonemal assembly factor 11(DNAAF11)
DNAAF2	55172	dynein axonemal assembly factor 2(DNAAF2)
DNAAF3	352909	dynein axonemal assembly factor 3(DNAAF3)
DNAAF4	161582	dynein axonemal assembly factor 4(DNAAF4)
DNAAF5	54919	dynein axonemal assembly factor 5(DNAAF5)
DNAAF6	139212	dynein axonemal assembly factor 6(DNAAF6)
DNAH1	25981	dynein axonemal heavy chain 1(DNAH1)
DNAH11	8701	dynein axonemal heavy chain 11(DNAH11)
DNAH5	1767	dynein axonemal heavy chain 5(DNAH5)
DNAI1	27019	dynein axonemal intermediate chain 1(DNAI1)
DNAI2	64446	dynein axonemal intermediate chain 2(DNAI2)
DNAJA1	3301	DnaJ heat shock protein family (Hsp40) member A1(DNAJA1)
DNAJB13	374407	DnaJ heat shock protein family (Hsp40) member B13(DNAJB13)
DNAJB5	25822	DnaJ heat shock protein family (Hsp40) member B5(DNAJB5)
DNAL1	83544	dynein axonemal light chain 1(DNAL1)
DNM2	1785	dynamin 2(DNM2)
DRC1	92749	dynein regulatory complex subunit 1(DRC1)
ECM1	1893	extracellular matrix protein 1(ECM1)

ECM2	1842	extracellular matrix protein 2(ECM2)
ELAVL2	1993	ELAV like RNA binding protein 2(ELAVL2)
EMP1	2012	epithelial membrane protein 1(EMP1)
ENG	2022	endoglin(ENG)
ENHO	375704	energy homeostasis associated(ENHO)
ENPP1	5167	ectonucleotide pyrophosphatase/phosphodiesterase 1(ENPP1)
ENTREP1	9413	endosomal transmembrane epsin interactor 1(ENTREP1)
EQTN	54586	equatorin(EQTN)
ERCC6L2	375748	ERCC excision repair 6 like 2(ERCC6L2)
EXOSC3	51010	exosome component 3(EXOSC3)
F13A1	2162	coagulation factor XIII A chain(F13A1)
F13B	2165	coagulation factor XIII B chain(F13B)
F2	2147	coagulation factor II, thrombin(F2)
F5	2153	coagulation factor V(F5)
FAM120A	23196	family with sequence similarity 120A(FAM120A)
FAM120AOS	158293	family with sequence similarity 120A opposite strand(FAM120AOS)
FAM166B	730112	family with sequence similarity 166 member B(FAM166B)
FAM205A	259308	SPATA31 subfamily F member 1(SPATA31F1)
FAM219A	203259	family with sequence similarity 219 member A(FAM219A)
FAM221B	392307	family with sequence similarity 221 member B(FAM221B)
FAM27D1	724094	family with sequence similarity 27 member D1(FAM27D1)
FANCC	2176	FA complementation group C(FANCC)
FANCG	2189	FA complementation group G(FANCG)
FBP1	2203	fructose-bisphosphatase 1(FBP1)
FBP2	8789	fructose-bisphosphatase 2(FBP2)
FBXO10	26267	F-box protein 10(FBXO10)
FETUB	26998	fetuin B(FETUB)
FGA	2243	fibrinogen alpha chain(FGA)
FGB	2244	fibrinogen beta chain(FGB)
FGD3	89846	FYVE, RhoGEF and PH domain containing 3(FGD3)
FGF7	2252	fibroblast growth factor 7(FGF7)
FGG	2266	fibrinogen gamma chain(FGG)
FLRT3	23767	fibronectin leucine rich transmembrane protein 3(FLRT3)
FN1	2335	fibronectin 1(FN1)
FOCAD	54914	focadhesin(FOCAD)
FOXB2	442425	forkhead box B2(FOXB2)
FOXD4L3	286380	forkhead box D4 like 3(FOXD4L3)
FOXD4L4	349334	forkhead box D4 like 4(FOXD4L4)
FOXD4L5	653427	forkhead box D4 like 5(FOXD4L5)
FOXD4L6	653404	forkhead box D4 like 6(FOXD4L6)
FOXE1	2304	forkhead box E1(FOXE1)

FREM1	158326	FRAS1 related extracellular matrix 1(FREM1)
FRMD3	257019	FERM domain containing 3(FRMD3)
FRMPD1	22844	FERM and PDZ domain containing 1(FRMPD1)
FST	10468	follistatin(FST)
FXN	2395	frataxin(FXN)
GABBR2	9568	gamma-aminobutyric acid type B receptor subunit 2(GABBR2)
GADD45G	10912	growth arrest and DNA damage inducible gamma(GADD45G)
GALT	2592	galactose-1-phosphate uridylyltransferase(GALT)
GAS1	2619	growth arrest specific 1(GAS1)
GAS2L2	246176	growth arrest specific 2 like 2(GAS2L2)
GAS8	2622	growth arrest specific 8(GAS8)
GBA2	57704	glucosylceramidase beta 2(GBA2)
GCNT1	2650	glucosaminyl (N-acetyl) transferase 1(GCNT1)
GDA	9615	guanine deaminase(GDA)
GKAP1	80318	G kinase anchoring protein 1(GKAP1)
GLIPR2	152007	GLI pathogenesis related 2(GLIPR2)
GLS	2744	glutaminase(GLS)
GNA14	9630	G protein subunit alpha 14(GNA14)
GNAQ	2776	G protein subunit alpha q(GNAQ)
GNE	10020	glucosamine (UDP-N-acetyl)-2-epimerase/N-acetylmannosamine kinase(GNE)
GNLY	10578	granulysin(GNLY)
GOLM1	51280	golgi membrane protein 1(GOLM1)
GPHN	10243	gephyrin(GPHN)
GRHPR	9380	glyoxylate and hydroxypyruvate reductase(GRHPR)
HABP2	3026	hyaluronan binding protein 2(HABP2)
HABP4	22927	hyaluronan binding protein 4(HABP4)
HACD4	401494	3-hydroxyacyl-CoA dehydratase 4(HACD4)
HAUS6	54801	HAUS augmin like complex subunit 6(HAUS6)
HEMGN	55363	hemogen(HEMGN)
HINT2	84681	histidine triad nucleotide binding protein 2(HINT2)
HLA-G	3135	major histocompatibility complex, class I, G(HLA-G)
HNRNPK	3190	heterogeneous nuclear ribonucleoprotein K(HNRNPK)
HOXA13	3209	homeobox A13(HOXA13)
HRCT1	646962	histidine rich carboxyl terminus 1(HRCT1)
HSD17B1	3292	hydroxysteroid 17-beta dehydrogenase 1(HSD17B1)
HSD17B3	3293	hydroxysteroid 17-beta dehydrogenase 3(HSD17B3)
HTR1A	3350	5-hydroxytryptamine receptor 1A(HTR1A)
HYDIN	54768	HYDIN axonemal central pair apparatus protein(HYDIN)
IARS1	3376	isoleucyl-tRNA synthetase 1(IARS1)
IDNK	414328	IDNK gluconokinase(IDNK)
IFI35	3430	interferon induced protein 35(IFI35)

IFI44	10561	interferon induced protein 44(IFI44)
IFI6	2537	interferon alpha inducible protein 6(IFI6)
IFNA1	3447	interferon alpha 13(IFNA13)
IFNA1	3439	interferon alpha 1(IFNA1)
IFNA10	3446	interferon alpha 10(IFNA10)
IFNA13	3447	interferon alpha 13(IFNA13)
IFNA14	3448	interferon alpha 14(IFNA14)
IFNA16	3449	interferon alpha 16(IFNA16)
IFNA17	3451	interferon alpha 17(IFNA17)
IFNA2	3440	interferon alpha 2(IFNA2)
IFNA21	3452	interferon alpha 21(IFNA21)
IFNA4	3441	interferon alpha 4(IFNA4)
IFNA5	3442	interferon alpha 5(IFNA5)
IFNA6	3443	interferon alpha 6(IFNA6)
IFNA7	3444	interferon alpha 7(IFNA7)
IFNA8	3445	interferon alpha 8(IFNA8)
IFNB1	3456	interferon beta 1(IFNB1)
IFNE	338376	interferon epsilon(IFNE)
IFNW1	3467	interferon omega 1(IFNW1)
IFT74	80173	intraflagellar transport 74(IFT74)
IGF1	3479	insulin like growth factor 1(IGF1)
IGF2	3481	insulin like growth factor 2(IGF2)
IGFBP1	3484	insulin like growth factor binding protein 1(IGFBP1)
IGFBP3	3486	insulin like growth factor binding protein 3(IGFBP3)
IGFBP6	3489	insulin like growth factor binding protein 6(IGFBP6)
IGFBPL1	347252	insulin like growth factor binding protein like 1(IGFBPL1)
IL11	3589	interleukin 11(IL11)
IL11RA	3590	interleukin 11 receptor subunit alpha(IL11RA)
IL12B	3593	interleukin 12B(IL12B)
IL16	3603	interleukin 16(IL16)
IL1B	3553	interleukin 1 beta(IL1B)
IL1RN	3557	interleukin 1 receptor antagonist(IL1RN)
IL20RA	53832	interleukin 20 receptor subunit alpha(IL20RA)
IL24	11009	interleukin 24(IL24)
IL5RA	3568	interleukin 5 receptor subunit alpha(IL5RA)
IL6	3569	interleukin 6(IL6)
IL9	3578	interleukin 9(IL9)
INHA	3623	inhibin subunit alpha(INHA)
IPPK	64768	inositol-pentakisphosphate 2-kinase(IPPK)
ISCA1	81689	iron-sulfur cluster assembly 1(ISCA1)
ITGB3	3690	integrin subunit beta 3(ITGB3)

ITGB4	3691	integrin subunit beta 4(ITGB4)
ITGB6	3694	integrin subunit beta 6(ITGB6)
IZUMO3	100129669	IZUMO family member 3(IZUMO3)
JAK2	3717	Janus kinase 2(JAK2)
KCNQ1	3784	potassium voltage-gated channel subfamily Q member 1(KCNQ1)
KIF24	347240	kinesin family member 24(KIF24)
KIF27	55582	kinesin family member 27(KIF27)
KIR2DL1	3802	killer cell immunoglobulin like receptor, two Ig domains and long cytoplasmic tail 1(KIR2DL1)
KIR2DL2	3803	killer cell immunoglobulin like receptor, two Ig domains and long cytoplasmic tail 2(KIR2DL2)
KLF9	687	KLF transcription factor 9(KLF9)
KLHL9	55958	kelch like family member 9(KLHL9)
KLK10	5655	kallikrein related peptidase 10(KLK10)
LAMA4	3910	laminin subunit alpha 4(LAMA4)
LGALS14	56891	galectin 14(LGALS14)
LGALS3	3958	galectin 3(LGALS3)
LIF	3976	LIF interleukin 6 family cytokine(LIF)
LINC00537	203274	long intergenic non-protein coding RNA 537(LINC00537)
LINC02872	401535	long intergenic non-protein coding RNA 2872(LINC02872)
LINGO2	158038	leucine rich repeat and Ig domain containing 2(LINGO2)
LMNA	4000	lamin A/C(LMNA)
LONP1	9361	lon peptidase 1, mitochondrial(LONP1)
LRRC19	64922	leucine rich repeat containing 19(LRRC19)
LRRC56	115399	leucine rich repeat containing 56(LRRC56)
LURAP1L	286343	leucine rich adaptor protein 1 like(LURAP1L)
MAMDC2	256691	MAM domain containing 2(MAMDC2)
MAP2	4133	microtubule associated protein 2(MAP2)
MAPRE3	22924	microtubule associated protein RP/EB family member 3(MAPRE3)
MBL2	4153	mannose binding lectin 2(MBL2)
MCIDAS	345643	multiciliate differentiation and DNA synthesis associated cell cycle protein(MCIDAS)
MELK	9833	maternal embryonic leucine zipper kinase(MELK)
MFSD14B	84641	major facilitator superfamily domain containing 14B(MFSD14B)
MGAT2	4247	alpha-1,6-mannosyl-glycoprotein 2-beta-N-acetylglucosaminyltransferase(MGAT2)
MGP	4256	matrix Gla protein(MGP)
MIR204	406987	microRNA 204(MIR204)
MIR23B	407011	microRNA 23b(MIR23B)
MIR24-1	407012	microRNA 24-1(MIR24-1)
MIR27B	407019	microRNA 27b(MIR27B)
MIR31	407035	microRNA 31(MIR31)
MIR7-1	407043	microRNA 7-1(MIR7-1)

MIRLET7A1	406881	microRNA let-7a-1(MIRLET7A1)
MIRLET7D	406886	microRNA let-7d(MIRLET7D)
MIRLET7F1	406888	microRNA let-7f-1(MIRLET7F1)
MLLT3	4300	MLLT3 super elongation complex subunit(MLLT3)
MMP11	4320	matrix metallopeptidase 11(MMP11)
MMP12	4321	matrix metallopeptidase 12(MMP12)
MMP15	4324	matrix metallopeptidase 15(MMP15)
MMP19	4327	matrix metallopeptidase 19(MMP19)
MMP26	56547	matrix metallopeptidase 26(MMP26)
MMP7	4316	matrix metallopeptidase 7(MMP7)
MOB3B	79817	MOB kinase activator 3B(MOB3B)
MPDZ	8777	multiple PDZ domain crumbs cell polarity complex component(MPDZ)
MPL	4352	MPL proto-oncogene, thrombopoietin receptor(MPL)
MSMP	692094	microseminoprotein, prostate associated(MSMP)
MTAP	4507	methylthioadenosine phosphorylase(MTAP)
MTHFR	4524	methylenetetrahydrofolate reductase(MTHFR)
MTMR14	64419	myotubularin related protein 14(MTMR14)
MUC4	4585	mucin 4, cell surface associated(MUC4)
MYF6	4618	myogenic factor 6(MYF6)
MYORG	57462	myogenesis regulating glycosidase (putative)(MYORG)
NAA35	60560	N-alpha-acetyltransferase 35, NatC auxiliary subunit(NAA35)
NANS	54187	N-acetylneuraminate synthase(NANS)
NCAM1	4684	neural cell adhesion molecule 1(NCAM1)
NCBP1	4686	nuclear cap binding protein subunit 1(NCBP1)
NCR1	9437	natural cytotoxicity triggering receptor 1(NCR1)
NCR2	9436	natural cytotoxicity triggering receptor 2(NCR2)
NCR3	259197	natural cytotoxicity triggering receptor 3(NCR3)
NDUFB6	4712	NADH: ubiquinone oxidoreductase subunit B6(NDUFB6)
NFIB	4781	nuclear factor I B(NFIB)
NFIL3	4783	nuclear factor, interleukin 3 regulated(NFIL3)
NFX1	4799	nuclear transcription factor, X-box binding 1(NFX1)
NINJ1	4814	ninjurin 1(NINJ1)
NME8	51314	NME/NM23 family member 8(NME8)
NMRK1	54981	nicotinamide riboside kinase 1(NMRK1)
NOL6	65083	nucleolar protein 6(NOL6)
NOL8	55035	nucleolar protein 8(NOL8)
NOS3	4846	nitric oxide synthase 3(NOS3)
NPR2	4882	natriuretic peptide receptor 2(NPR2)
NTRK2	4915	neurotrophic receptor tyrosine kinase 2(NTRK2)
NUDT2	318	nudix hydrolase 2(NUDT2)
NUTM2F	54754	NUT family member 2F(NUTM2F)

NUTM2G	441457	NUT family member 2G(NUTM2G)
NXNL2	158046	nucleoredoxin like 2(NXNL2)
ODAD1	93233	outer dynein arm docking complex subunit 1(ODAD1)
ODAD2	55130	outer dynein arm docking complex subunit 2(ODAD2)
ODAD3	115948	outer dynein arm docking complex subunit 3(ODAD3)
ODAD4	83538	outer dynein arm docking complex subunit 4(ODAD4)
OFD1	8481	OFD1 centriole and centriolar satellite protein(OFD1)
OGN	4969	osteoglycin(OGN)
OMD	4958	osteomodulin(OMD)
OR13J1	392309	olfactory receptor family 13 subfamily J member 1(OR13J1)
OR2S2	56656	olfactory receptor family 2 subfamily S member 2(OR2S2)
OSTF1	26578	osteoclast stimulating factor 1(OSTF1)
PABIR1	116224	PP2A Aalpha (PPP2R1A) and B55A (PPP2R2A) interacting phosphatase regulator 1(PABIR1)
PAEP	5047	progestagen associated endometrial protein(PAEP)
PAPPA	5069	pappalysin 1(PAPPA)
PAX5	5079	paired box 5(PAX5)
PCA3	50652	prostate cancer associated 3(PCA3)
PCDHA3	56145	protocadherin alpha 3(PCDHA3)
PCSK5	5125	proprotein convertase subtilisin/kexin type 5(PCSK5)
PGF	5228	placental growth factor(PGF)
PGM5	5239	phosphoglucomutase 5(PGM5)
PGR	5241	progesterone receptor(PGR)
PHF2	5253	PHD finger protein 2(PHF2)
PHF24	23349	PHD finger protein 24(PHF24)
PIF1	80119	PIF1 5'-to-3' DNA helicase(PIF1)
PIGO	84720	phosphatidylinositol glycan anchor biosynthesis class O(PIGO)
PIP5K1B	8395	phosphatidylinositol-4-phosphate 5-kinase type 1 beta(PIP5K1B)
PLAA	9373	phospholipase A2 activating protein(PLAA)
PLIN2	123	perilipin 2(PLIN2)
PLK1	5347	polo like kinase 1(PLK1)
POLR1E	64425	RNA polymerase I subunit E(POLR1E)
POMC	5443	proopiomelanocortin(POMC)
PRKACG	5568	protein kinase cAMP-activated catalytic subunit gamma(PRKACG)
PRLR	5618	prolactin receptor(PRLR)
PRSS3	5646	serine protease 3(PRSS3)
PRSS47P	138652	serine protease 47, pseudogene(PRSS47P)
PRUNE2	158471	prune homolog 2 with BCH domain(PRUNE2)
PRXL2C	195827	peroxiredoxin like 2C(PRXL2C)
PSAT1	29968	phosphoserine aminotransferase 1(PSAT1)
PSIP1	11168	PC4 and SRSF1 interacting protein 1(PSIP1)
PTAR1	375743	protein prenyltransferase alpha subunit repeat containing 1(PTAR1)

PTCH1	5727	patched 1(PTCH1)
PTGIS	5740	prostaglandin I2 synthase(PTGIS)
PTHLH	5744	parathyroid hormone like hormone(PTHLH)
PTPDC1	138639	protein tyrosine phosphatase domain containing 1(PTPDC1)
RASEF	158158	RAS and EF-hand domain containing(RASEF)
RBP4	5950	retinol binding protein 4(RBP4)
RECK	8434	reversion inducing cysteine rich protein with kazal motifs(RECK)
REXO4	57109	REX4 homolog, 3'-5' exonuclease(REXO4)
RFK	55312	riboflavin kinase(RFK)
RGP1	9827	RGP1 homolog, RAB6A GEF complex partner 1(RGP1)
RMI1	80010	RecQ mediated genome instability 1(RMI1)
RMRP	6023	RNA component of mitochondrial RNA processing endoribonuclease(RMRP)
RNF38	152006	ring finger protein 38(RNF38)
ROR2	4920	receptor tyrosine kinase like orphan receptor 2(ROR2)
RORB	6096	RAR related orphan receptor B(RORB)
RPGR	6103	retinitis pigmentosa GTPase regulator(RPGR)
RPP25L	138716	ribonuclease P/MRP subunit p25 like(RPP25L)
RPS6	6194	ribosomal protein S6(RPS6)
RRAGA	10670	Ras related GTP binding A(RRAGA)
RSPH1	89765	radial spoke head component 1(RSPH1)
RSPH3	83861	radial spoke head 3(RSPH3)
RSPH4A	345895	radial spoke head component 4A(RSPH4A)
RSPH9	221421	radial spoke head component 9(RSPH9)
RUSC2	9853	RUN and SH3 domain containing 2(RUSC2)
RYR1	6261	ryanodine receptor 1(RYR1)
S1PR3	1903	sphingosine-1-phosphate receptor 3(S1PR3)
S1PR3	1903	sphingosine-1-phosphate receptor 3(S1PR3)
SAXO1	158297	stabilizer of axonemal microtubules 1(SAXO1)
SDF2L1	23753	stromal cell derived factor 2 like 1(SDF2L1)
SECISBP2	79048	SECIS binding protein 2(SECISBP2)
SEMA4D	10507	semaphorin 4D(SEMA4D)
SERPINB3	6317	serpin family B member 3(SERPINB3)
SERPINB4	6318	serpin family B member 4(SERPINB4)
SERPINC1	462	serpin family C member 1(SERPINC1)
SERPINE1	5054	serpin family E member 1(SERPINE1)
SH3GL2	6456	SH3 domain containing GRB2 like 2, endophilin A1(SH3GL2)
SHB	6461	SH2 domain containing adaptor protein B(SHB)
SHC3	53358	SHC adaptor protein 3(SHC3)
SIGMAR1	10280	sigma non-opioid intracellular receptor 1(SIGMAR1)
SIT1	27240	signaling threshold regulating transmembrane adaptor 1(SIT1)
SLC24A2	25769	solute carrier family 24 member 2(SLC24A2)

SLC25A51	92014	solute carrier family 25 member 51(SLC25A51)
SLC28A3	64078	solute carrier family 28 member 3(SLC28A3)
SLC35D2	11046	solute carrier family 35 member D2(SLC35D2)
SMC5	23137	structural maintenance of chromosomes 5(SMC5)
SMU1	55234	SMU1 DNA replication regulator and spliceosomal factor(SMU1)
SNAPC3	6619	small nuclear RNA activating complex polypeptide 3(SNAPC3)
SPAG1	6674	sperm associated antigen 1(SPAG1)
SPAG5	10615	sperm associated antigen 5(SPAG5)
SPAG8	26206	sperm associated antigen 8(SPAG8)
SPATA31A1	647060	SPATA31 subfamily A member 1(SPATA31A1)
SPATA31A3	727830	SPATA31 subfamily A member 3(SPATA31A3)
SPATA31A5	727905	SPATA31 subfamily A member 5(SPATA31A5)
SPATA31A6	389730	SPATA31 subfamily A member 6(SPATA31A6)
SPATA31A7	26165	SPATA31 subfamily A member 7(SPATA31A7)
SPATA31C1	441452	SPATA31 subfamily C member 1(SPATA31C1)
SPATA31C2	645961	SPATA31 subfamily C member 2(SPATA31C2)
SPATA31D1	389763	SPATA31 subfamily D member 1(SPATA31D1)
SPATA31D3	389762	SPATA31 subfamily D member 3(SPATA31D3)
SPATA31D4	389761	SPATA31 subfamily D member 4(SPATA31D4)
SPATA31E1	286234	SPATA31 subfamily E member 1(SPATA31E1)
SPIN1	83985	SPNS lysolipid transporter 1, lysophospholipid(SPNS1)
SPIN1	10927	spindlin 1(SPIN1)
SPINK4	27290	serine peptidase inhibitor Kazal type 4(SPINK4)
SPTLC1	10558	serine palmitoyltransferase long chain base subunit 1(SPTLC1)
SST	6750	somatostatin(SST)
STK36	27148	serine/threonine kinase 36(STK36)
STOML2	30968	stomatin like 2(STOML2)
SUSD3	203328	sushi domain containing 3(SUSD3)
SYCP3	50511	synaptonemal complex protein 3(SYCP3)
SYK	6850	spleen associated tyrosine kinase(SYK)
TAF1L	138474	TATA-box binding protein associated factor 1 like(TAF1L)
TBC1D2	55357	TBC1 domain family member 2(TBC1D2)
TDRD7	23424	tudor domain containing 7(TDRD7)
TEK	7010	TEK receptor tyrosine kinase(TEK)
TESK1	7016	testis associated actin remodelling kinase 1(TESK1)
TFRC	7037	transferrin receptor(TFRC)
TGFB1	7040	transforming growth factor beta 1(TGFB1)
TGFBI	7045	transforming growth factor beta induced(TGFBI)
TGFBR1	7046	transforming growth factor beta receptor 1(TGFBR1)
THPO	7066	thrombopoietin(THPO)
TIMP2	7077	TIMP metallopeptidase inhibitor 2(TIMP2)

TIMP3	7078	TIMP metallopeptidase inhibitor 3(TIMP3)
TJP2	9414	tight junction protein 2(TJP2)
TLE1	7088	TLE family member 1, transcriptional corepressor(TLE1)
TLE4	7091	TLE family member 4, transcriptional corepressor(TLE4)
TLN1	7094	talin 1(TLN1)
TMC1	117531	transmembrane channel like 1(TMC1)
TMEM215	401498	transmembrane protein 215(TMEM215)
TMEM252	169693	transmembrane protein 252(TMEM252)
TMEM8B	51754	transmembrane protein 8B(TMEM8B)
TMOD1	7111	tropomodulin 1(TMOD1)
TNFRSF1A	7132	TNF receptor superfamily member 1A(TNFRSF1A)
TNFSF10	8743	TNF superfamily member 10(TNFSF10)
TNFSF13	8741	TNF superfamily member 13(TNFSF13)
TNR	7143	tenascin R(TNR)
TOMM5	401505	translocase of outer mitochondrial membrane 5(TOMM5)
TOPORS	10210	TOP1 binding arginine/serine rich protein, E3 ubiquitin ligase(TOPORS)
TPM2	7169	tropomyosin 2(TPM2)
TRAF1	7185	TNF receptor associated factor 1(TRAF1)
TRIM14	9830	tripartite motif containing 14(TRIM14)
TRMO	51531	tRNA methyltransferase O(TRMO)
TRMT10B	158234	tRNA methyltransferase 10B(TRMT10B)
TRPM3	80036	transient receptor potential cation channel subfamily M member 3(TRPM3)
TRPM6	140803	transient receptor potential cation channel subfamily M member 6(TRPM6)
TSTD2	158427	thiosulfate sulfurtransferase like domain containing 2(TSTD2)
TTC39B	158219	tetratricopeptide repeat domain 39B(TTC39B)
TUSC1	286319	tumor suppressor candidate 1(TUSC1)
TUT7	79670	terminal uridylyl transferase 7(TUT7)
TYMS	7298	thymidylate synthetase(TYMS)
TYRP1	7306	tyrosinase related protein 1(TYRP1)
UBAP1	51271	ubiquitin associated protein 1(UBAP1)
UBAP2	55833	ubiquitin associated protein 2(UBAP2)
UBE2R2	54926	ubiquitin conjugating enzyme E2 R2(UBE2R2)
UBQLN1	29979	ubiquilin 1(UBQLN1)
UNC13B	10497	unc-13 homolog B(UNC13B)
VCP	7415	valosin containing protein(VCP)
VPS13A	23230	vacuolar protein sorting 13 homolog A(VPS13A)
WNK2	65268	WNK lysine deficient protein kinase 2(WNK2)
WRN	7486	WRN RecQ like helicase(WRN)
XIST	7503	X inactive specific transcript(XIST)
XPA	7507	XPA, DNA damage recognition and repair factor(XPA)
ZBTB5	9925	zinc finger and BTB domain containing 5(ZBTB5)

ZCCHC7	84186	zinc finger CCHC-type containing 7(ZCCHC7)
ZDHHC21	340481	zinc finger DHHC-type palmitoyltransferase 21(ZDHHC21)
ZFAND5	7763	zinc finger AN1-type containing 5(ZFAND5)
ZMYND10	51364	zinc finger MYND-type containing 10(ZMYND10)
ZNF169	169841	zinc finger protein 169(ZNF169)
ZNF367	195828	zinc finger protein 367(ZNF367)
ZNF484	83744	zinc finger protein 484(ZNF484)
ZNF510	22869	zinc finger protein 510(ZNF510)
ZNF658	26149	zinc finger protein 658(ZNF658)
ZNF782	158431	zinc finger protein 782(ZNF782)
ZNG1C	445571	Zn regulated GTPase metalloprotein activator 1C(ZNG1C)
ZNG1F	644019	Zn regulated GTPase metalloprotein activator 1F(ZNG1F)

List of potential gene of abortion and miscarriage relate gene based in ClinVar, Disgenet, Monarch database

Supplementary Table S2. Details of Sanger sequencing for polymerase chain reaction

position	Gene	Name	Primer sequence (5' -> 3')
chr2:233274367	ALPG	rs575918099_F	ACTGAAACTGAACCCTCCAA
		rs575918099_R	CCAGCAGAGGAAGCAACG
chr2:238283646	COL6A3	rs116238578_F	ACGTCCTGCTTGTTCATGTA
		rs116238578_R	AATTGCTGCTCTGATGTCCT
chr3:195488449	MUC4	rs200737893_F	GCACAGATTCCTCAGACCTT
		rs200737893_R	GGCCAACCTCCCACTCTG
chr10:115334164	HABP2	rs542838125_F	AGACACATGATTCCTCCTGC
		rs542838125_R	ATACACTCCGCTCTGACCTT
chr17:34074883	GAS2L2	rs140842796_F	CTGTACTGTGCCCAACTACT
		rs140842796_R	ATGTGATGGTACGTGTAGGG
chr19:10897280	DNM2	rs763894364_F	CTGTCTCTCAAGGCTGTGTG
		rs763894364_R	TGAGTGGACGAGTGATGAGT
chr20:48130848	PTGIS	rs13306027_F	TGCCAGGTGTGTGAAGATAG
		rs13306027_R	CTTGCAGGGGAATATGGGTC
chr20:52789538	CYP24A1	rs114476330_F	CGCCTCTTCACAATTTCCAG
		rs114476330_R	GTATGGCAAGATTTTCCGCA
chr16:16276686	ABCC6	rs758166222_F	GGTGAGAGGTGGAGAGAATG
		rs758166222_R	TGCGTGTCCAGAATAAACCT
chr16:16276686	ABCC6	rs527236047_F	CTTCCCTCTCCTCTGCAAAT
		rs527236047_R	TGGCCGAGAATGCTATGAAT
